# Drug-Drug Interactions Between COVID-19 Treatments and Psychotropic Medications: An Updated Study

**DOI:** 10.7759/cureus.50469

**Published:** 2023-12-13

**Authors:** Ujwal Boppana, Thomas S Leonard, Ayodeji Jolayemi, Maliha I Ansari, Andrew Salib

**Affiliations:** 1 Psychiatry, Interfaith Medical Center, Brooklyn, USA; 2 College of Medicine, Pramukhswami Medical College, Anand, IND; 3 College of Medicine, Florida International University, Herbert Wertheim College of Medicine, Florida, USA; 4 College of Medicine, American University of Antigua College of Medicine, St. John’s, ATG

**Keywords:** benzodiazepines, mood stabilizers, antidepressants, antipsychotics, medication therapy management, psychotropic, drug-drug interactions, covid 19

## Abstract

The recent evolution of coronavirus disease 2019 (COVID-19) treatments has created challenges for healthcare providers in terms of new potential interactions between these COVID-19 treatments and psychotropic drugs in patients with psychiatric disorders. Current clinical practice guidelines on managing interactions between psychotropic medications and COVID-19 treatments do not account for the newer COVID-19 medications. There is a need for updated patient management recommendations that take into account drug interactions between psychotropic drugs and the latest pharmacological approaches to COVID-19 treatment. A search of literature pertaining to drug interactions and outcomes in patients concurrently prescribed COVID-19 treatments and psychotropic medications was conducted. Drug databases were also analyzed to screen for interactions. Our review focuses on the most recent and effective COVID-19 treatments, including Paxlovid^TM^ (nirmatrelvir/ritonavir), remdesivir, dexamethasone, tocilizumab, and baricitinib.

The study provides condensed and easily interpretable tables for healthcare providers to screen for potentially harmful drug interactions. We discuss the implications of our findings on appropriate treatment plan selection by healthcare providers for patients taking select antipsychotics, antidepressants, mood stabilizers, and benzodiazepines while receiving COVID-19 treatments. Notably, Paxlovid^TM ^may interact with several medications, particularly antipsychotics and anxiolytics, necessitating close monitoring and, in some cases, reconsideration of use. We find that dexamethasone, remdesivir, tocilizumab, and baricitinib have fewer reported interactions with psychotropics, and while some monitoring is necessary, no major adjustments are recommended for their administration in conjunction with psychotropic medications. These findings underscore the importance of careful consideration and monitoring when combining COVID-19 treatments with other medications to mitigate the risk of adverse interactions and ensure patient safety.

## Introduction and background

Patients with coronavirus disease 2019 (COVID-19) often present with comorbidities, including psychiatric disorders such as schizophrenia spectrum disorders, bipolar spectrum disorders, anxiety disorders, and major depressive disorders. Many of these individuals are on maintenance treatment for their psychiatric conditions, but the stress and isolation associated with COVID-19 can lead to an exacerbation of psychiatric symptoms, necessitating medication management adjustments or additional psychiatric interventions [1]. Moreover, in some cases, COVID-19 can induce new-onset psychiatric manifestations, including delirium, mood disturbances, anxiety, and psychosis, which may require treatment with antipsychotics such as haloperidol and sedatives such as lorazepam [1]. These observations align with scientific research and clinical experience, underscoring the intricate interplay between COVID-19 and mental health and emphasizing the importance of individualized patient assessments in guiding treatment decisions [1,2].

Psychotropic medications may interact with medical treatments for COVID-19, potentially exacerbating adverse effects and influencing the course and outcome of the underlying medical condition [3]. To address this concern, numerous studies have investigated the interactions between psychotropics and COVID-19 medications [3-5]. However, it is essential to acknowledge several noteworthy limitations inherent in these investigations. Firstly, these studies have primarily examined a restricted subset of psychotropic medications, thus omitting several widely used psychotropics in clinical practice, including lurasidone, oxcarbazepine, topiramate, lamotrigine, temazepam, fluphenazine, and ziprasidone. This limitation raises concerns about the comprehensiveness of the findings and necessitates further exploration into the interactions involving these specific psychotropics. Secondly, the dynamic nature of COVID-19 treatments has significantly evolved over the past one to two years, leading to a notable shift in the medications employed to manage the infection. For instance, treatments such as hydroxychloroquine and azithromycin, which were once considered, are no longer recommended for COVID-19 management. Furthermore, the most current guidelines for COVID-19, as of January 2022, now incorporate a combination therapy involving nirmatrelvir and ritonavir [6,7].

Regrettably, these evolving aspects have not been adequately addressed in previous review articles investigating the interactions between psychotropics and COVID-19 medications [3-5,8]. The prevalence rates of various psychiatric symptoms during the COVID-19 epidemic, such as an overall prevalence of depressive symptoms (depression) at 23.9%, anxiety symptoms (anxiety) at 23.4%, stress at 14.2%, distress at 16.0%, insomnia symptoms at 26.5%, post-traumatic stress symptoms (PTSS) at 24.9%, and poor mental health at 19.9% [1]. Many patients with underlying psychiatric disorders may be infected with COVID-19, and up to 25% of patients affected by COVID-19 were shown to experience new-onset psychiatric manifestations [3]. These statistics provide insights into the mental health impact of the COVID-19 pandemic, highlighting the strong comorbidity between COVID-19 and psychiatric illness and the importance of consideration of drug interactions in the concurrent management of these conditions.

The authors performed a literature search using updated COVID-19 treatment regimens to identify novel interactions between psychotropics and COVID-19 treatments. Our objective was to leverage the latest evidence on pharmacological interactions to furnish valuable guidance for the prudent and effective administration of psychotropic medications in individuals with psychiatric conditions undergoing treatment for COVID-19.

## Review

Methods

A literature search was conducted with search terms COVID-19 treatment AND (Sars-CoV-2 treatment OR Coronavirus Disease-19 treatment OR Sars Coronavirus 2 treatment OR Coronavirus 2019 treatment) through Google Scholar and PubMed databases. National Institute of Health (NIH), Centers for Disease Control and Prevention (CDC), Wolters Kluwer’s UpToDate, and Merck Manual were also consulted to determine the currently recommended COVID-19 treatments to consider for interaction with psychotropic medication. Paxlovid (nirmatrelvir/ritonavir), dexamethasone, remdesivir, tocilizumab, and baricitinib were determined to be the recommended COVID-19 treatments to consider for interactions with psychotropics. Previously reported COVID-19 treatments, such as hydroxychloroquine, were not considered due to newer guidelines detailing their lack of efficacy. Psychotropic medications to consider for drug interaction were selected by determining the most commonly used medications in the four major categories of psychopharmacology, antipsychotics, antidepressants, mood stabilizers, and benzodiazepines.

The next step was to review the drug-drug interactions between the aforementioned COVID-19 medications and commonly used psychotropic medications in patients with COVID-19. The search strategy used the syntax: COVID-19 AND (psychotropics OR antipsychotics OR antidepressants OR mood stabilizers OR benzodiazepines OR Paxlovid OR dexamethasone OR remdesivir OR tocilizumab OR baricitinib). Additional drug interaction databases, namely Lexicomp, Micromedex, and Epocrates, were used to construct tables summarizing known interactions between selected drugs using data such as interaction type, severity, and management recommendations.

Two researchers independently conducted the screening, data extraction, and quality assessment processes, with any discrepancies resolved through a consensus-driven approach and the involvement of two additional researchers. This methodological strategy was implemented to ensure a rigorous and transparent synthesis of evidence concerning drug interactions between psychotropic medications and COVID-19 treatments. The integration of both quantitative and qualitative methods is pursued to achieve a comprehensive understanding of the subject matter.

The qualitative analysis focused on data derived from case studies, case reports, and pharmacological studies pertaining to drug interactions. Parameters such as study type, drug interactions, clinical outcomes, changes in management, and sample size were systematically extracted by the researchers. A narrative synthesis was then crafted to elucidate common mechanisms through which these interactions manifested.

In addition to the aforementioned sources, data on drug interactions was sourced from reputable drug databases, including Lexicomp, Micromedex, and Epocrates. A systematic categorization approach was adopted: interactions flagged as severe by one or more databases were labeled as "contraindicated," while those identified as having moderate interactions by two or more databases were characterized as requiring "therapy modification" or "monitoring." The absence of reported drug interactions across all databases led to the characterization of the interaction as "no known interaction." This meticulous process enhances the reliability and validity of our findings, contributing to a robust analysis of drug interactions in the context of psychotropic medications and COVID-19 treatments.

**Figure 1:**
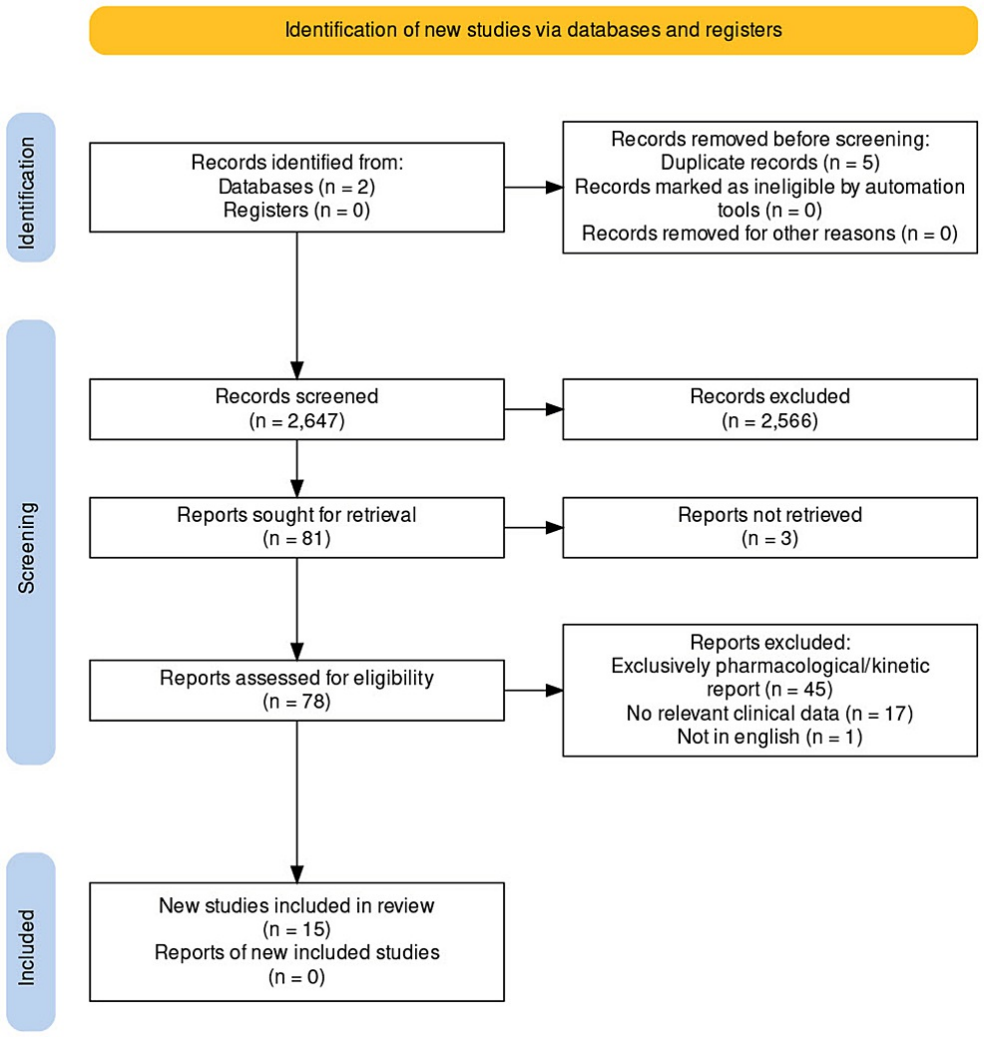
PRISMA flowchart for the study selection process PRISMA: Preferred Reporting Items for Systematic Reviews and Meta-Analyses.

Inclusion criteria were set to filter for review, case reports/series, and randomized controlled studies that reported on clinical events and outcomes (Figure 1). Exclusion criteria were set to those that focused solely on pharmacodynamics/kinetics and those that did not report clinically relevant outcomes. Articles older than 10 years were excluded. Articles published before 2013 were excluded.

Data extracted from reviewed articles included diagnosis, clinical outcomes, drug interaction, interaction type, and intervention. Suggestions (e.g., consider a switch, monotherapy) based on the articles reviewed were also summarized and used in recommendations for managing medication interactions. A summary of the findings from the literature on drug-drug interactions is shown in Table 1.

**Table 1:** Summary of included studies COVID-19: Coronavirus disease 2019; CYP: Cytochrome P450; DDI: Drug-drug interaction. Paxlovid = Paxlovid^TM^

Authors	Year	Title	Study Type	Notes/Conclusions
Gatti et al. [3]	2021	Clinically significant drug interactions between psychotropic agents and repurposed COVID-19 therapies.	Review	The article examines psychotropic medication interactions with COVID-19 antiretrovirals (e.g., lopinavir/ritonavir and darunavir/cobicistat), highlighting risks in mild-to-moderate COVID-19 and noting therapy suspensions in severe cases with ICU admission. It emphasizes monitoring, dose adjustments, and research, especially regarding remdesivir, for patients with mental health conditions and COVID-19.
Plasencia-Garcia et al. [4]	2021	Drug-drug interactions between COVID-19 treatments and antipsychotic drugs: integrated evidence from 4 databases and a systematic review.	Review	This article examines antipsychotic interactions with COVID-19 drugs, categorizing them as safe (e.g., remdesivir, baricitinib, anakinra), cautious (clozapine, baricitinib, favipiravir with specific antipsychotics), and concerning (chloroquine, hydroxychloroquine, azithromycin, lopinavir/ritonavir). Tocilizumab may affect antipsychotic metabolism, while olanzapine appears relatively safer with lopinavir/ritonavir. Consider potential QT prolongation and CYP interactions when prescribing antipsychotics to COVID-19 patients, following safety guidelines.
Marzolini et al. [9]	2022	Recommendations for the management of drug-drug interactions between the COVID-19 antiviral nirmatrelvir/ritonavir (Paxlovid) and comedications.	Review	Used Liverpool drug interaction tool to screen for DDIs Paxlovid and an extensive list of psychotropic and non-psychotropic medication. Discussed pharmacological and pharmacokinetic basis for DDIs with Paxlovid.
Azanza et al. [10]	2022	Interactions listed in the Paxlovid fact sheet, classified according to risks, pharmacological groups, and consequences.	Brief Report	Briefly summarized DDIs with Paxlovid from various drug classes (non-psychotropic medication as well). Gives recommendation for clinical adjustment required for given DDI.
Plasencia-García et al. [11]	2022	Drug-drug interactions between COVID-19 treatments and antidepressants, mood stabilizers/anticonvulsants, and benzodiazepines: integrated evidence from 3 databases.	Review	Utilized Lexicomp, micromedex, and Liverpool Drug Interaction Group to screen for interactions between hydroxychloroquine, chloroquine, azithromycin, lopinavir-ritonavir, remdesivir, favipiravir, tocilizumab, baricitinib, anakinra, and dexamethasone and an extensive list of psychotropic medications. Also included case reports published pre-covid of DDI with similar mechanisms. Stratified DDIs and shortlisted drugs that were contraindicated for concurrent use with select COVID-19 therapies.
Shah et al. [12]	2021	Benzodiazepine interaction with COVID-19 drugs.	Brief Report	This report examines benzodiazepine interactions with COVID-19 drugs like Lopinavir/ritonavir and atazanavir, intensifying effects due to ritonavir's enzyme inhibition. Pairing midazolam, triazolam, or alprazolam with atazanavir or ritonavir can cause sedation, confusion, respiratory depression, and adverse effects, emphasizing the preference for temazepam, oxazepam, or lorazepam over alprazolam.
Yalcin and Allegaert [13]	2022	COVID-19 and antiepileptic drugs: an approach to guide practices when nirmatrelvir/ritonavir is co-prescribed.	Review	Authors used drug interaction checkers such as Lexicomp in conjunction with pharmacokinetic modeling to propose dose adjustments for drug interactions between nirmatrelvir/ritonavir and select psychotropic medications.
Bonaccorso et al. [14]	2021	Clozapine, neutropenia and COVID-19: should clinicians be concerned? 3 months report.	Case series	In summary, the study found a significant drop in neutrophil count in psychiatric inpatients on clozapine with COVID-19. Clinicians should closely monitor neutrophil counts in these patients, considering the potential interaction between clozapine and COVID-19.
Butler et al. [15]	2020	Clozapine prescribing in COVID-19 positive medical inpatients: a case series [15]	Case series	This retrospective case series examines continuing clozapine in COVID-19 patients, involving eight cases, with four halting clozapine during hospitalization. While some required intensive care due to COVID-19 pneumonia, clozapine was generally considered safe. Delirium was common, and one case experienced psychosis relapse post-clozapine discontinuation. Further, larger-scale research is required for confirmation.
Liu et al. [16]	2022	Neutropenia after the coadministration of clozapine and nirmatrelvir-ritonavir in a patient with SARS-CoV-2 infection: A case report with a literature review.	Case Report	This case report details a clozapine-treated patient with SARS-CoV-2 infection, experiencing neutropenia after taking Paxlovid, valproic acid, and clozapine. Neutropenia, potentially life-threatening, likely resulted from interactions involving Paxlovid, SARS-CoV-2 infection, valproic acid, fluvoxamine, and clozapine, especially during concurrent SARS-CoV-2 infection and Paxlovid use.
Ghasemiyeh et al. [17]	2021	Psychiatric adverse drug reactions and potential anti-COVID-19 drug interactions with psychotropic medications.	Literature Review	This review assessed COVID-19 treatment interactions with psychotropic drugs, focusing on mechanisms and management, including drugs like azithromycin, hydroxychloroquine, chloroquine, convalescent plasma, famotidine, interferon, and ivermectin. Major interactions, mainly pharmacokinetic via cytochrome enzymes, were analyzed to prevent adverse effects.
Ostuzzi et al. [18]	2020	Safety of psychotropic medications in people with COVID-19: Evidence review and practical recommendations.	Review	The review highlighted interactions between COVID-19 drugs (e.g., chloroquine, hydroxychloroquine, azithromycin, ribavirin, lopinavir/ritonavir) and psychiatric medications, impacting QTc interval, hepatotoxicity, concentration, and myelosuppression. Remdesivir has fewer interactions, except with St. John's Wort and specific anticonvulsants. Favipiravir poses lower interaction risks. Tocilizumab can alleviate depressive symptoms but carries hepatotoxicity risks.
Mohebbi et al. [19]	2020	Drug interactions of psychiatric and COVID-19 medications.	Review	Psychiatric medication could be used safely in combination with COVID-19 pharmacotherapy if medications were selected with the least possibility of interaction and also careful monitoring. Some important points: concomitant use of SSRIs with antiviral medications and/or chloroquine/hydroxychloroquine increases the risk of hypoglycemia and the concomitant use of pimozide or midazolam with antiviral medications is contraindicated.
Izci and Kulacaoglu [5]	2020	Drug interactions between COVID-19 and psychiatric medications: A mini review.	Review	Chloroquine, hydroxychloroquine, azithromycin, ribavirin, and lopinavir/ritonavir interact with psychiatric drugs, impacting QTc interval, hepatotoxicity, myelosuppression, and concentrations. Remdesivir has fewer interactions, except with St. John's Wort and specific anticonvulsants. Favipiravir has lower interaction risks and fewer psychiatric medication side effects. Tocilizumab is generally safe with psychiatric drugs, improving depressive symptoms but posing hepatotoxicity risk.
Bilbul et al. [20]	2020	Psychopharmacology of COVID-19.	Review	Certain medications like chloroquine, hydroxychloroquine, and azithromycin can prolong the QT interval, posing risks for cardiac patients. When using antipsychotics for delirium, be cautious, especially in cardiac patients; consider alternatives like alpha-2 agonists or antiepileptics for high-risk cases. Melatonin helps with sleep-wake disturbances, while benzodiazepines should be a last resort for highly agitated patients. In anxious patients with respiratory distress, carefully weigh the use of low-dose benzodiazepines.

Results

Drug-Drug Interactions: Antipsychotics and COVID-19 Treatments

Paxlovid^TM^, a combination of the antiviral drugs nirmatrelvir and ritonavir, is a CYP3A4 substrate and also inhibits CYP3A4 [9]. This can result in potentially harmful interactions when used in combination with certain antipsychotics, as they too are heavily metabolized by CYP3A4 [9]. Interactions have been reported with lurasidone and should be avoided, while the use of Paxlovid^TM^ with aripiprazole [21], brexpiprazole [22], cariprazine [23], Iloperidone [24], and quetiapine [25] should be reconsidered and closely monitored. Haloperidol [26], clozapine [27], olanzapine [28], and risperidone [27] are also metabolized by CYP3A4 and should be closely monitored, but can be considered for combination therapy if the patient's drug levels and health are closely monitored. On the other hand, no known interactions have been reported between Paxlovid^TM^ and chlorpromazine, fluphenazine, loxapine, perphenazine, trifluoperazine, asenapine [29], or paliperidone.

Remdesivir, an antiviral that inhibits viral RNA polymerase, and tocilizumab, a monoclonal antibody that inhibits interleukin-6 (IL-6) receptors, have not shown any known interactions with antipsychotics based on data from various databases [5]. However, tocilizumab may have potential hepatotoxic side effects when used in combination with clozapine [5]. In addition, there are some reports that tocilizumab may decrease the serum concentration of CYP3A4 substrates [4]. As a result of the findings in Table 2, Table 3 provides a summary of recommendations for the combinations of antipsychotics and medications used to treat COVID-19.

**Table 2:** The interactions between medications for COVID-19 and antipsychotic medications COVID-19: Coronavirus disease 2019; CYP3A4: Cytochrome P450 3A4; CYP: Cytochrome P450. Paxlovid = Paxlovid^TM^

Psychotropic Drug	Paxlovid^TM^	Tocilizumab	Remdesivir	Baricitinib	Dexamethasone
Haloperidol	Paxlovid increases Haloperidol concentration through CYP3A4 inhibition. Monitoring for extrapyramidal symptoms, sedation, and QT interval should be considered [9,26].	May decrease the serum concentration of CYP3A4 substrates [4].	No interactions reported	No interactions reported	No interactions reported
Lurasidone	Paxlovid increases Lurasidone concentration through CYP3A4 inhibition. Pharmacokinetic studies report lurasidone AUC concentrations to increase 9-fold when coadministered with strong CYP3A4 inhibitors. Co-administration is contraindicated.	May decrease the serum concentration of CYP3A4 substrates [4].	No interactions reported	No interactions reported	No interactions reported
Quetiapine	Paxlovid increases Quetiapine concentration through CYP3A4 inhibition. Monitor for weight gain, sedation, confusion, respiratory depression. Dosage adjustment should be considered [25].	May decrease the serum concentration of CYP3A4 substrates [4].	No interactions reported	No interactions reported	No interactions reported
Olanzapine	Paxlovid decreases Olanzapine concentration through induction of CYP1A2- and/or glucuronosyltransferase-mediated olanzapine metabolism [28]. Monitor for efficacy and dose adjustment.	No interactions reported	No interactions reported	No interactions reported	No interactions reported
Clozapine	Paxlovid increases clozapine concentration through interaction with CYP3A4, CYP2D6, and CYP1A2 [27]. Clinical significance is not clear, and therapy should be closely monitored for toxicity and dose reduction.	Potential hepatotoxic side effects when used in combination [5].	No interactions reported	Baricitinib and clozapine are both myelosuppressive agents, warranting close monitoring for signs of severe immunosuppression. No pharmacokinetic interactions were reported.	Dexamethasone decreases clozapine concentration through weak CYP3A4 induction. Monitor for efficacy and dose adjustment.

**Table 3:** Recommendations for combinations of COVID-19 medications and antipsychotics (X) – Avoid combination, (D) – Consider therapy modification, (C) – Monitor therapy, (A) – No known interaction. COVID-19: Coronavirus disease 2019; CYP3A4: Cytochrome P450 3A4; CYP: Cytochrome P450.

		Nirmatelvir/Ritonavir (Paxlovid^TM^)	Remdesivir	Dexamethasone	Tocilizumab	Baricitinib
		First-generation (typical) antipsychotics	Chlorpromazine	A	A	A	A	A
Fluphenazine	A		A	A	A	A
Haloperidol	C - CYP3A4		A	A	A	A
Loxapine	A		A	A	A	A
Perphenazine	A		A	A	A	A
Thioridazine	D - CYP2D6		A	A	A	A
Trifluoperazine	A		A	A	A	A
Second-generation (atypical) antipsychotics	Aripiprazole	D - CYP3A4	A	A	A	A
	Asenapine	A	A	A	A	A
	Brexpiprazole	D - CYP3A4	A	A	A	A
	Cariprazine	D - CYP3A4	A	A	A	A
	Clozapine	C - CYP3A4	A	C - CYP3A4 inducer	C - IL-6 inhibitor-related CYP induction	C - Myelosuppressive agent
	Iloperidone	D - CYP3A4	A	A	A	A
	Lurasidone	X - CYP3A4	A	A	A	A
	Olanzapine	C - CYP1A2	A	A	A	A
	Paliperidone	A	A	A	A	A
	Quetiapine	D - CYP3A4	A	A	A	A
	Risperidone	C - P-glycoprotein/ABCB1 Inhibitors	A	A	A	A
	Ziprasidone	C - CYP3A4	A	A	A	A

Drug-Drug Interactions: Antidepressants and COVID-19 Treatments

Based on information from various databases, there are no known interactions reported between remdesivir and most antidepressants. However, it is not recommended to use remdesivir in combination with St. John's Wort as St. John's Wort may lower the plasma levels of remdesivir [5]. Similarly, no known interactions have been reported between tocilizumab and most antidepressants [5]. Paxlovid^TM^ is a medication used for the treatment of COVID-19, and its use with certain antidepressants warrants close monitoring due to potential interactions [10]. Specifically, the use of Paxlovid^TM^ with desvenlafaxine should be monitored closely due to the potential risk of reduced substrate concentrations, which could result in treatment inefficacy through CYP3A4 inhibition [11]. Similarly, the use of Paxlovid^TM^ with trazodone [30] should be reconsidered, monitored, and potentially reduced in dosage due to the potential risk of increased substrate concentrations, which could lead to toxicity through CYP3A4 inhibition. The use of Paxlovid^TM^ with mirtazapine should also be closely monitored due to the potential risk of increased substrate concentrations, which could result in toxicity through CYP3A4 inhibition [11]. 

However, according to data from various databases, no known interactions have been reported between Paxlovid^TM^ and several commonly used antidepressants, including fluoxetine, sertraline, paroxetine, fluvoxamine, citalopram, venlafaxine, duloxetine, and vortioxetine [30]. Additionally, there are no known interactions between tocilizumab and antidepressants based on data from various databases [5]. Similarly, no known interactions have been reported between baricitinib and antidepressants. A summary of some of the reported interactions between some antidepressants and COVID-19 medications, as reported in the literature, is shown in Table 4.

**Table 4:** Interactions between medications for COVID-19 and antidepressant medications COVID-19: Coronavirus disease 2019; CYP3A4: Cytochrome P450 3A4; CYP: Cytochrome P450. Paxlovid = Paxlovid^TM^

Psychotropic Drug	Paxlovid^TM^	Tocilizumab	Remdesivir	Baricitinib	Dexamethasone
Mirtazapine	Paxlovid increases mirtazapine concentration through CYP3A4 inhibition. Monitor for toxicity and dose adjustment [11].	No interactions reported	No interactions reported	No interactions reported	No interactions reported
Bupropion	Paxlovid decreases bupropion concentration through CPY2B6 induction. Monitor for efficacy and dose adjustment [11].	No interactions reported	No interactions reported	No interactions reported	No interactions reported
Trazodone	Paxlovid increases trazodone concentration through CYP3A4 inhibition. Monitor for toxicity and dose adjustment [11].	No interactions reported	No interactions reported	No interactions reported	No interactions reported
Fluoxetine	No interactions reported	No interactions reported	No interactions reported	No interactions reported	No interactions reported
Sertraline	No interactions reported	No interactions reported	No interactions reported	No interactions reported	No interactions reported
Paroxetine	No interactions reported	No interactions reported	No interactions reported	No interactions reported	No interactions reported

On the basis of the findings from the literature, recommendations for combining antidepressants and COVID-19 medications are listed in Table 5.

**Table 5:** Recommendations on the combination of medications for COVID-19 and antidepressants (X) – Avoid combination, (D) – Consider therapy modification, (C) – Monitor therapy, (B) – No action needed, (A) – No known interaction. COVID-19: Coronavirus disease 2019; CYP3A4: Cytochrome P450 3A4.

		Nirmatelvir/Ritonavir (Paxlovid^TM^)	Remdesivir	Dexamethasone	Tocilizumab	Baricitinib
		SSRI's	Fluoxetine	A	A	A	A	A
Sertraline	A		A	A	A	A
Paroxetine	A		A	A	A	A
Fluvoxamine	A		A	A	A	A
Citalopram	A		A	A	A	A
SNRI's	Venlafaxine	A	A	A	A	A
	Desvenlafaxine	B - CYP3A4 Increased desvenlafaxine concentration	A	A	A	A
	Duloxetine	A	A	A	A	A
NRDI's	Bupropion	C - Decreased buproprion concentration	A	A	A	A
Other	Trazodone	D - CYP3A4 Increased trazodone concentration	A	A	A	A
	Mirtazapine	C - CYP3A4 Increased mirtazapine concentration	A	A	A	A
	Vortioxetine	A	A	A	A	A

Drug-Drug Interactions: Mood Stabilizers and COVID-19 Treatments

Paxlovid^TM^ is a medication used for the treatment of COVID-19, and its use with certain mood stabilizers warrants close monitoring due to potential interactions. Specifically, Paxlovid^TM^ is contraindicated with carbamazepine due to the risk of increased exposure to carbamazepine and reduced Paxlovid^TM^ concentrations through CYP3A4 inhibition [31]. On the other hand, no known interactions have been reported between Paxlovid^TM^ and several other mood stabilizers, including gabapentin, oxcarbazepine, pregabalin, topiramate, lithium, and zonisamide [31].

The use of Paxlovid^TM^ with lamotrigine [32] and valproic acid [12] should be closely monitored due to the potential risk of reduced serum concentrations of these antiepileptic medications resulting in reduced seizure prophylaxis. However, based on available data from various databases, no known interactions have been reported between baricitinib and mood stabilizers [30]. A summary of some of the reported interactions between some mood stabilizers and COVID-19 medications, as reported in the literature, is shown in Table 5.

**Table 6:** The interactions between medications for COVID-19 and mood stabilizers COVID-19: Coronavirus disease 2019; CYP3A4: Cytochrome P450 3A4. Paxlovid = Paxlovid^TM^

Psychotropic Drug	Paxlovid^TM^	Tocilizumab	Remdesivir	Baricitinib	Dexamethasone
Carbamazepine	Paxlovid inhibits CYP3A4, increasing carbamazepine concentration, while carbamazepine induces CYP3A4, reducing Paxlovid efficacy. Co-administration is contraindicated due to reduced Paxlovid effectiveness and potential carbamazepine toxicity [31].	No interactions reported	No interactions reported	No interactions reported	Carbamazepine induces CYP3A4, reducing dexamethasone concentration. Consider dose adjustment [30].
Lamotrigine	Paxlovid reduces lamotrigine concentration via enhanced glucuronidation. Monitor and adjust the dose as needed [32].	No interactions reported	No interactions reported	No interactions reported	No interactions reported
Valproate	Paxlovid may slightly lower Valproate concentration with weak evidence. Consider monitoring and potential dose adjustment [12].	No interactions reported	No interactions reported	No interactions reported	No interactions reported
Lithium	No interactions reported	No interactions reported	No interactions reported	No interactions reported	Potential increase in concentration of lithium [33].

On the basis of the findings from the literature, recommendations for combining mood stabilizers and COVID-19 medications are listed in Table 6.

**Table 7:** Recommendations for combining COVID-19 medications and mood stabilizers (X) – Avoid combination, (D) – Consider therapy modification, (C) – Monitor therapy, (B) – No action needed, (A) – No known interaction. COVID-19: Coronavirus disease 2019; CYP3A4: Cytochrome P450 3A4.

		Nirmatelvir/Ritonavir (Paxlovid^TM^)	Remdesivir	Dexamethasone	Tocilizumab	Baricitinib
		Mood Stabilizers	Carbamazepine	X - CYP3A4 Increased carbamazepine concentration	B - Reduced remdesivir concentration	D - CYP3A4 decreased dexamethasone concentration	A	A
Gabapentin	A		A	A	A	A
Lamotrigine	C - glucorinidation decreased lamotrigine concentration		A	A	A	A
Oxcarbazepine	A		A	A	A	A
Pregabalin	A		A	A	A	A
Topiramate	A		A	A	A	A
Valproic acid	C - multiple mechanisms decreased valproic acid concentration		A	A	A	A
Lithium	A		A	A	A	A
Zonisamide	A		A	A	A	A

Drug-Drug Interactions: Benzodiazepines and COVID-19 Treatments

Paxlovid^TM^ is a medication used for the treatment of COVID-19, and its use with certain anxiolytics warrants close monitoring due to potential interactions. The use of Paxlovid^TM^ with alprazolam should be reconsidered and closely monitored due to the potential risk of increased substrate concentrations, which could result in toxicity through CYP3A4 inhibition [34]. Conversely, no known interactions have been reported between Paxlovid^TM^ and several other anxiolytics [3], including lorazepam, temazepam, oxazepam, and quazepam. However, Paxlovid^TM^ is contraindicated with midazolam [33] and triazolam [1] due to the risk of increased substrate concentrations with the potential for toxicity through CYP3A4 inhibition. The use of Paxlovid^TM^ with diazepam, clonazepam, chlordiazepoxide, flurazepam, and clorazepate should also be closely monitored due to the potential risk of increased substrate concentrations through CYP3A4 inhibition [30]. On the other hand, based on available data from various databases, no known interactions have been reported between baricitinib and anxiolytics [30]. It is worth noting that the use of dexamethasone with midazolam and triazolam should also be closely monitored [30]. A summary of some of the reported interactions between benzodiazepines and COVID-19 medications, as reported in the literature, is shown in Table 8.

**Table 8:** The interactions between medications for COVID-19 and mood stabilizers CYP3A4: Cytochrome P450 3A4; COVID-19: Coronavirus disease 2019. Paxlovid = Paxlovid^TM^

Psychotropic Drug	Paxlovid^TM^	Tocilizumab	Remdesivir	Baricitinib	Dexamethasone
Midazolam	Paxlovid significantly increases the concentration of midazolam through the inhibition of CYP3A enzymes [33]. Co-administration is contraindicated.	No interactions reported	No interactions reported	No interactions reported	Dexamethasone may reduce the concentration of midazolam through induction of CYP3A4
Alprazolam	Paxlovid increases the concentration of alprazolam through CYP3A4 inhibition. Dose adjustment should be considered [34].	No interactions reported	No interactions reported	No interactions reported	No interactions reported
Diazepam	Paxlovid can increase or decrease the concentration of diazepam through CYP3A4 inhibition of CYP2C19 induction [11]. Monitor for efficacy/toxicity and dose adjustment.	No interactions reported	No interactions reported	No interactions reported	No interactions reported
Clonazepam	Paxlovid increases the concentration of alprazolam through CYP3A4 inhibition [11].	No interactions reported	No interactions reported	No interactions reported	No interactions reported
Chlordiazepoxide	Paxlovid increases the concentration of alprazolam through CYP3A4 inhibition [11].	No interactions reported	No interactions reported	No interactions reported	No interactions reported
Lorazepam	No interactions reported	No interactions reported	No interactions reported	No interactions reported	No interactions reported

On the basis of the findings from the literature, recommendations for combining benzodiazepines and COVID-19 medications are listed in Table 9.

**Table 9:** Recommendations for combining benzodiazepines and COVID-19 medications. (X) – Avoid combination, (D) – Consider therapy modification, (C) – Monitor therapy, (B) – No action needed, (A) – No known interaction. CYP3A4: Cytochrome P450 3A4; COVID-19: Coronavirus disease 2019.

		Nirmatelvir/Ritonavir (Paxlovid^TM^)	Remdesivir	Dexamethasone	Tocilizumab	Baricitinib
		Benzodiazepines	Alprazolam	D - CYP3A4 inhibition - Increased substrate concentrations	A	A	A	A
Diazepam	C - CYP3A4 inhibition - Increased substrate concentrations		A	A	A	A
Lorazepam	A		A	A	A	A
Clonazepam	C - CYP3A4 inhibition - Increased substrate concentrations		A	A	A	A
Temazepam	A		A	A	A	A
Chlordiazepoxide	C - CYP3A4 inhibition - Increased substrate concentrations		A	A	A	A
Oxazepam	A		A	A	A	A
Flurazepam	C - CYP3A4 inhibition - Increased substrate concentrations		A	A	A	A
Clorazepate	C - CYP3A4 inhibition - Increased substrate concentrations		A	A	A	A
Quazepam	A		A	A	A	A
Midazolam	X - CYP3A4 inhibition - Increased substrate concentrations		A	B - CYP3A4 induction - reduced substrate concentrations	A	A
Triazolam	X - CYP3A4 inhibition - Increased substrate concentrations		A	B - CYP3A4 induction - reduced substrate concentrations	A	A

Discussion

The rapid development of treatment regimens for the management of COVID-19 warrants a careful examination of drug interactions. Co-administration of certain psychotropic medications with COVID-19 treatments is a particularly important point of concern as many psychotropics already have narrow therapeutic indices [4]. Tables 2, 4, 6, 8 summarize the pharmacological effects of drug interactions, while Tables 3, 5, 7, 9 summarize interactions and recommendations for selected combinations of psychotropics and COVID-19 treatments. Providers can use the tables in the study to help guide treatment plans in the following manner: while interactions labeled X in Tables 3, 5, 7, 9 should always be avoided, D, C, and B require more careful considerations of costs and benefits. If alternative therapies with no known interactions (class A) are available, they should be considered as substitutes for combinations of drugs labeled D and C. 

**Figure 2:**
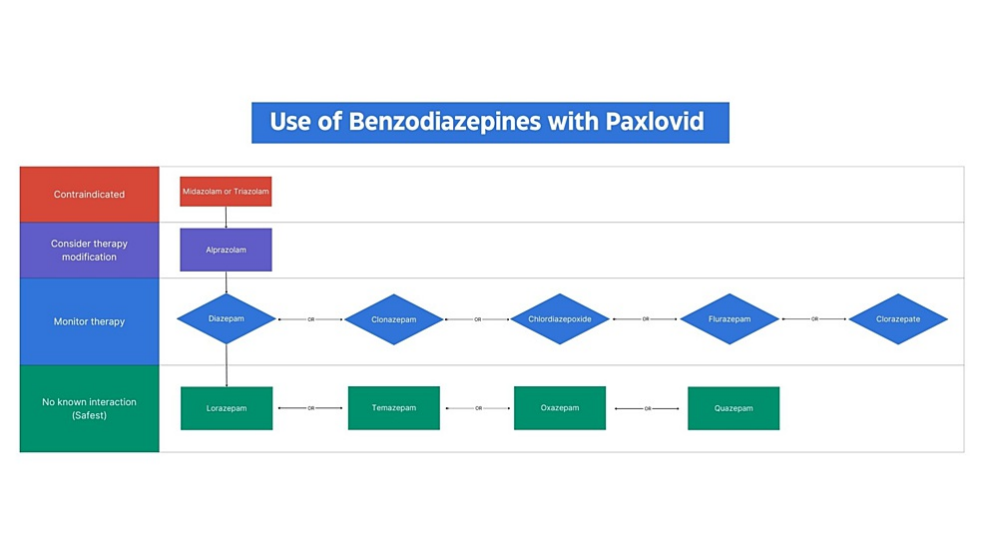
An illustrative guide for the interpretation of the concurrent use of benzodiazepines and Paxlovid Using Table 9 as an example, this figure serves as an illustrative guide for the interpretation of the concurrent use of benzodiazepines and Paxlovid^TM^.

Figure 2 stands as an illustrative model for the construction of a decision flow chart based on the information derived from any of the tables. In this instance, the focus is on benzodiazepines, highlighting contraindications with midazolam and triazolam (labeled X in Table 9) and emphasizing the need for identifying alternative therapies. For patients on benzodiazepines, especially in light of potential interactions highlighted in Table 8, precision in navigating the therapeutic protocol is crucial. The flowchart facilitates a seamless transition to benzodiazepines like lorazepam, temazepam, oxazepam, and quazepam, known for their safety with Paxlovid^TM^. This systematic approach offers a clear pathway for medication adjustments, minimizing potential risks associated with drug interactions. For example, if a patient is currently prescribed midazolam and requires Paxlovid^TM^, the recommended transition is to lorazepam, ensuring a seamless continuum of care with minimized risks. It is essential to emphasize that medications within the same row of the flowchart are considered comparable in terms of interaction risk. While the flowchart provides valuable guidance, clinical judgment remains paramount. Medical professionals should tailor their decisions based on individual patient cases, considering nuanced factors and specific needs to optimize patient outcomes.

Consistent with previous publications on interactions between ritonavir-containing drugs and psychotropics, Paxlovid^TM^, has numerous drug interactions with varying classes of psychotropics primarily through inhibition of cytochrome P450 enzymes [12]. Co-administration of Paxlovid^TM^ with drugs such as lurasidone, carbamazepine, midazolam, and triazolam should always be avoided and is categorized as X. Co-administration with drugs such as quetiapine and trazodone should warrant consideration of alternative therapies or dose modifications and is categorized as D.

Interactions categorized as C, such as co-administration of Paxlovid^TM^ with clozapine, olanzapine, risperidone, bupropion, lamotrigine, and diazepam, warrant close monitoring. One case report detailed an occurrence of neutropenia after co-administration of clozapine and Paxlovid^TM^ and advised close monitoring of white blood cell and neutrophil counts when co-administering these medications [16]. Additional case series of clozapine use in COVID-19 patients support its use with close monitoring of absolute neutrophil counts and serum clozapine levels [15]. Considering the many interactions antipsychotics have with antivirals such as Paxlovid^TM^, our inquiry revealed that first-generation antipsychotics such as fluphenazine and atypical antipsychotics such as paliperidone are relatively safe to co-administer [10]. Although antidepressants do undergo metabolism through the cytochrome P450 systems, certain drugs such as sertraline and fluoxetine do not have significant reported drug interactions with Paxlovid^TM^ [11]. Benzodiazepines, such as lorazepam, oxazepam, and temazepam, that rely on glucuronidation as opposed to cytochrome P450 as the mode of elimination are reported in drug databases to have fewer interactions with Paxlovid^TM^ and are consistent with previous literature on the subject [12]. Paxlovid^TM^ co-administration with mood stabilizers such as gabapentin, topiramate, lithium, and zonisamide has no reported drug interactions [11]. As such, these drugs should be considered as alternatives for patients administered Paxlovid^TM^ concurrently with drugs such as lamotrigine or valproic acid [20].

The remaining COVID-19 treatments, dexamethasone, remdesivir, tocilizumab, and baricitinib have fewer reported interactions [9]. Dexamethasone is both metabolized by CYP3A4 and induces the enzyme [3]. Co-administration of dexamethasone with drugs such as clozapine may reduce serum concentrations of clozapine and should be monitored for dose modification. Conversely, co-administration of dexamethasone with CYP3A4 inducers such as carbamazepine can reduce dexamethasone exposure and should prompt consideration of alternative therapies. Consistent with previous literature [4], there are no reported interactions between remdesivir, tocilizumab and baricitinib and the psychotropic medications discussed in this study. Clozapine and baricitinib co-administration, which is cautioned due to both agents having immunosuppressive effects [4], there are no evidence-based adjustments to be made to clinical practice in the administration of remdesivir, tocilizumab, and baricitinib to patients receiving psychotropic medications.

The pharmacological mechanisms of aforementioned drug interactions can be generalised into interactions that involve inhibition of cytochrome enzymes, increasing concentrations of drugs metabolised through this pathway and induction of cytochrome enzymes that reduces concentrations of drugs metabolised through the said pathway. The COVID-19 treatment with the greatest number of interactions was Paxlovid^TM^, commonly involved CYP3A4, across multiple drug classes. Close monitoring of adverse effects of the drug concurrently administered with Paxlovid^TM^ should be considered as the serum concentration of these can be elevated due to CYP3A4 inhibition. For antipsychotics such as haloperidol, clinicians should monitor for extrapyramidal symptoms, sedation, confusion, and QT prolongation [9,26]. Co-administration of Paxlovid^TM^ with benzodiazepines can increase serum concentration resulting in symptoms of benzodiazepine toxicity such as altered mental status [11]. The induction of cytochrome enzymes is another major mechanism of drug interaction between COVID-19 medications and psychotropics. Paxlovid-related induction of CYP-1A2 has the potential to decrease olanzapine levels, resulting in subtherapeutic dosing [28]. Carbamazepine-related induction of CYP-3A4 can result in increased metabolism of dexamethasone, resulting in reduced corticosteroid exposure, potentially warranting an increase in dexamethasone dosing [30].

Limitations

The length of time used to study drug interactions, especially for the newer COVID-19 medications, may be too short to observe longer-term interactions that may be significant. In addition, given the epidemiology of COVID-19, the sample sizes of the subset of patients with psychiatric disorders in some instances may not be large enough to draw useful conclusions about drug-drug interactions.

## Conclusions

In conclusion, the COVID-19 pandemic has posed numerous challenges to healthcare providers, including managing patients with COVID-19 and comorbidities, such as those requiring psychotropic medications. Understanding potential interactions between COVID-19 treatments and psychotropic medications is crucial for optimizing patient outcomes. This discussion has highlighted the potential interactions between Paxlovid^TM^, remdesivir, tocilizumab, and baricitinib and commonly used psychotropic medications such as antipsychotics, antidepressants, mood stabilizers, and anxiolytics. Healthcare providers should be aware of these potential interactions and closely monitor patients for adverse effects. Using the tables provided in the study providers will be able to easily find information regarding potentially dangerous interactions and adjust their treatment plans accordingly. As new data and treatments become available, ongoing vigilance and collaboration among healthcare providers will be essential for managing the complex needs of patients with COVID-19 and comorbidities.
